# The microbiota-gut-brain axis in depression: unraveling the relationships and therapeutic opportunities

**DOI:** 10.3389/fimmu.2025.1644160

**Published:** 2025-09-25

**Authors:** Zhangcheng Zhu, Yiwen Cheng, Xia Liu, Xiaocui Xu, Wenwen Ding, Zongxin Ling, Jiaming Liu, Guangyong Cai

**Affiliations:** ^1^ Department of Preventive Medicine, School of Public Health and Management, Wenzhou Medical University, Wenzhou, Zhejiang, China; ^2^ State Key Laboratory for Diagnosis and Treatment of Infectious Diseases, National Clinical Research Center for Infectious Diseases, China-Singapore Belt and Road Joint Laboratory on Infection Research and Drug Development, National Medical Center for Infectious Diseases, Collaborative Innovation Center for Diagnosis and Treatment of Infectious Diseases, The First Affiliated Hospital, Zhejiang University School of Medicine, Hangzhou, Zhejiang, China; ^3^ Yuhang Institute for Collaborative Innovation and Translational Research in Life Sciences and Technology, Hangzhou, Zhejiang, China; ^4^ Department of Intensive Care Unit, The First Affiliated Hospital, School of Medicine, Zhejiang University, Hangzhou, Zhejiang, China; ^5^ Department of Anesthesiology, Affiliated Hospital of Nantong University, Nantong, Jiangsu, China; ^6^ Lishui Key Laboratory of Brain Health and Severe Brain Disorders, Department of Rehabilitation, Lishui Second People’s Hospital, Lishui, Zhejiang, China

**Keywords:** depression, gut microbiota, microbiota-gut-brain axis, neuroinflammation, therapeutic interventions

## Abstract

Depression, a highly prevalent and relapsing mental disorder, exacts profound personal and socioeconomic tolls globally, warranting urgent scientific and clinical attention. Emerging evidence from both preclinical models and human clinical investigations has established the microbiota-gut-brain axis (MGBA) as a critical determinant in depression pathogenesis. This intricate bidirectional network integrates gut microbiota with central nervous system function, influencing mental health through mechanisms previously underrecognized. This review systematically synthesizes gut microbiota alterations associated with depression and their impacts on neuroendocrine, neuroimmune, and metabolic pathways. Advanced therapeutic strategies targeting the MGBA are discussed, including probiotics, fecal microbiota transplantation, and artificial intelligence-enabled microbiome interventions for depression management. While challenges in standardization, mechanistic understanding, efficacy and safety remain, MGBA-centered approaches offer a promising shift toward microbiota-based diagnostics and personalized treatments for depression.

## Introduction

Depression, particularly major depressive disorder (MDD), is a debilitating mental health condition marked by persistent low mood, loss of interest in activities, cognitive impairments, and suicidal tendencies, significantly impacting patients’ social functioning and quality of life (1–3). In 2008, the World Health Organization (WHO) identified MDD as the third leading cause of global disease burden (4). The prevalence of MDD continues to rise, with approximately 280 million individuals worldwide affected each year, including 700,000 deaths by suicide (5). Notably, the COVID-19 pandemic further exacerbated this issue, with increased social isolation, economic stress, and health-related anxieties contributing to a significant rise in depression rates, placing additional strain on both societal and familial structures (6, 7). Despite the widespread use of first-line antidepressants such as selective serotonin reuptake inhibitors (e.g., fluoxetine, sertraline) and serotonin-norepinephrine reuptake inhibitors (e.g., venlafaxine, duloxetine), these medications often show delayed efficacy (typically 4–6 weeks) and may cause adverse side effects including sexual dysfunction, weight gain, and gastrointestinal disturbances. Evidence indicates that over one-third of patients have a poor or minimal response to these treatments (8), highlighting the incomplete understanding of depression’s underlying pathophysiology and the urgent need for alternative therapeutic targets.

The therapeutic challenge in treating MDD has sparked growing interest in the microbiota-gut-brain axis (MGBA), a potential mechanism for both understanding the disorder and exploring new interventions. The MGBA concept evolved over decades of research, from early discoveries of gut-brain hormonal interactions to the current view of the gut microbiome as a key regulator of neuropsychiatric health (9, 10). The human gut microbiota, made up of trillions of microorganisms (11), acts as a “second brain,” influencing the central nervous system (CNS) through multiple pathway (12). Dysbiosis of gut microbiota refers to significant changes in the quantity and function of gut microbiota, which can significantly affect host physiology through MGBA, contributing to disorders like Parkinson’s disease, autism, bipolar disorder, and schizophrenia (13–15). In MDD, both clinical and preclinical studies show microbial composition changes during depressive states (16–18). Fecal microbiota transplantation (FMT) from depressed individuals can induce depression-like behaviors in animals (19–22), while certain probiotics have shown promise in alleviating depressive symptoms (23–26). This growing body of evidence emphasizes the potential of microbiota-based interventions in managing MDD.

As research into the gut microbiota’s role in depression expands, new potential targets and mechanisms are emerging. Current studies have identified three key pathways through which gut microbiota influence depression via the MGBA: immune regulation (e.g., cytokine release) (27–29), endocrine modulation (e.g., hypothalamic-pituitary-adrenal (HPA) axis activity) (30, 31), and neural signaling (e.g., vagus nerve communication and neurotransmitter regulation) (32, 33). This evolving MGBA framework highlights the diagnostic and therapeutic potential of gut microbiota in managing depression, positioning microbiota-targeted interventions as a promising avenue for antidepressant development. This review explores the contributions of MGBA to MDD pathogenesis, focusing on key regulatory nodes in the immuno-neuro-endocrine network, and also discusses innovative treatment strategies that utilize gut microbiota modulation, showcasing their potential for next-generation depression therapies.

## Gut dysbiosis and depression

Gut microbiota has emerged as a crucial factor in the development and progression of depression, with increasing evidence supporting its influence on mental health through various mechanisms. Research in both preclinical and clinical studies has shown that gut microbiota can modulate brain function and behavior through MGBA. In preclinical studies, the gut microbiota has been found to affect neurotransmitter production, inflammation, and stress response systems, all of which are involved in depression. Animal models have demonstrated that altering the gut microbiota can lead to changes in mood and behavior, mimicking symptoms of depression or showing improvements with specific microbiota compositions. Clinical studies have further reinforced these findings, showing that individuals with depression often have an altered gut microbiome compared to healthy controls. Additionally, Moreover, interventions aimed at modifying the gut microbiota—such as probiotics, prebiotics, or FMT—have shown promising effects in alleviating depressive symptoms in some patients. These studies suggest that gut microbiota may play a significant role in depression and could offer new therapeutic avenues for managing the condition.

### Preclinical studies

The pivotal role of gut microbiota in the pathophysiology of psychiatric disorders, such as MDD, has become increasingly recognized (34–37). However, the precise mechanisms through which the microbiota influences mental health remain unclear. As outlined in in **Table 1**, a key focus of current research involves investigating the dynamic interplay between changes in microbial communities and psychiatric phenotypes through animal models. This approach has become central in the field, seeking to elucidate the underlying processes connecting gut microbiota to mental health disorders.

**Table 1:** Changes in gut microbiota in different models of depression.

Model	Changed gut microbiota	Main findings	Ref
CUMS mice	*Parasutterella* and *Akkermansia*↑, Bifidobacteriaceae and Lactobacillaceae↓	The gut microbiota can impact anxiety and depression-like behaviors in mice through the microbiota-inflammation-brain axis.	(20)
CUMS mice FMT mice	*Helicobacter*, *Bacteroides*, and *Desulfovibrio*↑, *Lactobacillus*, *Bifidobacterium*, and *Akkermansia*↓	Stressed animals’ gut microbiota can activate microglia in the hippocampal dentate gyrus. Modulating the microbiome or inhibiting microglial activation may effectively reduce stress sensitivity.	(64)
CSDS mice	Bacilli, Bacteroidia, Mollicutes, and Verrucomicrobiae classes↑, Erysipelotrichi class↓	Heat-sterilized *Bifidobacterium breve* can alter gut microbiota composition in CSDS mice, preventing depression-like behaviors and IL-1β expression.	(48)
Chronic immobilization stress mice	Bacteroidetes and Proteobacteria ↑, Firmicutes ↓ Rumen *Clostridium*, anaerobic bacteria↓, *Prevotella*, *Desulfovibrio* ↑	*Prevotella* may influence the release of inflammatory factors through the production of short-chain fatty acids, potentially contributing to depression.	(65)
MS mice	*Bifidobacterium*, *Lactobacillus*, *Clostridium parvum*, *Clostridium coccoides*↑	Maternal-infant separation stress disrupts gut microbiome composition, activates neuroimmune responses in the hippocampus, and induces inflammatory factors, contributing to depression-like behavior.	(53)
MS mice	Microbial diversity↓ *Lactobacillus reuteri*↓	MS induces gut microbiota dysbiosis, notably reducing *L. reuteri* abundance. Supplementing with *L. reuteri* improves neurobehavioral abnormalities in MS mice by enhancing intestinal amino acid transport and restoring synaptic plasticity in the mPFC.	(55)
LH mice	Lactic acid levels were positively correlated with *Lactobacillus* abundance, *Lactobacillus*, *Clostridioides* cluster III, and *Anaerofustis*↑	Altered gut microbiota composition, particularly *Lactobacillus*, significantly affects mice’s susceptibility and resistance to inescapable electric shock stress.	(57)
LH rat	Clostridiales↓	The microbiota distribution in LH rats significantly differed from control rats, contributing to the onset of IBS.	(58)
Corticosterone -induced depression	Microbial diversity↓ *Lactobacillus vaginalis*↑ unclassified *Bifidobacterium*↑	Corticosterone regulates ceramide levels by altering gut microbiota composition, inducing mitochondrial dysfunction and contributing to depression onset.	(61)
Nonhuman primate model of depression	The depressive-like monkeys had characteristic disturbances of Firmicutes.	The gut microbiome is involved in depression-like behavior by regulating glycerophospholipid metabolism.	(63)

(The ↑/↓ are shown as P<0.05).

CSDS, Chronic social defeat stress; CUMS, Chronic unpredictable mild stress; FMT, Fecal microbiota transplantation; IBS, Irritable bowel syndrome; LH, Learned helplessness; MS, Maternal separation.

A widely employed animal model in this research is the chronic unpredictable mild stress (CUMS) model, which induces stress-related behaviors by exposing animals to a series of unpredictable, mild stressors, mimicking the symptoms of depression (35, 38–41). In mice subjected to CUMS, significant alterations in the gut microbiota occur, including an increase in *Proteobacteria* and *Verrucomicrobia*, coupled with a decrease in beneficial bacteria such as *Bifidobacteriaceae* and *Lactobacillaceae*. Notably, FMT from CUMS mice to healthy recipients successfully replicates depressive phenotypes (20), establishing a direct link between gut dysbiosis and depression. Furthermore, these depressive-like behaviors can be reversed by probiotic interventions, which operate through three synergistic mechanisms: (1) enhancing serotoninergic neurotransmission by promoting 5-HT synthesis and TPH expression, (2) suppressing neuroinflammation via IDO inhibition, and (3) normalizing hyperactivity of the HPA axis (42). Similarly, microbial metabolites such as short-chain fatty acids (SCFAs), including butyrate and propionate, have proven effective in alleviating CUMS-induced behavioral deficits, reinforcing the therapeutic potential of microbiota-targeted strategies (43–45).

The chronic social defeat stress (CSDS) model is another frequently used animal model to study depression, specifically targeting psychological and social stressors that contribute to depressive behaviors. In this model, mice are subjected to repeated episodes of social subjugation by a dominant conspecific, inducing severe psychological stress that leads to persistent behavioral changes. These behaviors typically include social withdrawal and anhedonia, both defining features of depression (46). Recent research has highlighted the connection between CSDS-induced depression-like behaviors and alterations in the gut microbiota, offering valuable insights into the underlying mechanisms. Mice exposed to CSDS display significant gut microbiota dysbiosis, characterized by reduced alpha diversity and a notable decline in *Lactobacillus* abundance (47). Additionally, studies by Aika Kosuge et al. have identified shifts in the abundance of bacterial classes, including an increase in Bacilli, Bacteroidia, Mollicutes, and Verrucomicrobiae, which are linked to metabolic and immune-modulatory functions. These microbial changes may influence the stress response and contribute to depressive symptoms. Conversely, the reduction in Erysipelotrichi, a class associated with inflammation and metabolic disturbances, further underscores the role of gut microbiota in modulating depression-like behaviors (48). Therapeutic interventions targeting specific bacterial strains have demonstrated efficacy in alleviating depression-like behaviors in CSDS mice, suggesting that microbiota modulation could serve as a promising therapeutic avenue for depression treatment (49, 50).

The maternal separation (MS) model, which involves early deprivation of pup-mother interactions, induces core depressive phenotypes such as anhedonia, reduced exploratory behavior, and diminished interest during developmental maturation. This model is widely used to simulate the long-term impact of early-life stress on neurodevelopment and subsequent behavioral outcomes (51, 52). Emerging studies have shown that MS results in significant changes in gut microbiota composition in mice (53, 54), which appear to mediate the neuropsychiatric effects associated with early-life stress. Notably, oral administration of multi-strain probiotic formulations has demonstrated significant improvements in anxiety and depressive symptoms in MS-exposed mice, suggesting that gut microbiota dysbiosis plays a pivotal role in the pathophysiology of MS-induced neuropsychiatric symptoms. This could be mediated through gut-brain axis and neuroimmune signaling pathways (55, 56). The growing body of evidence emphasizes the profound impact of early-life stressors on both the gut microbiome and brain, opening new possibilities for therapeutic interventions aimed at modulating the gut microbiota to alleviate mood and anxiety disorders.

The learned helplessness (LH) model is a widely recognized animal model for studying depression. It consists of two phases: initially, animals are subjected to inescapable stress, followed by a re-exposure to escapable stress, during which they demonstrate a marked reduction in escape responses. Studies show that LH mice experience a significant decrease in gut microbial diversity, with a notable reduction in beneficial bacteria such as Lactobacillaceae (57) and Clostridiales incertae sedis (58). This disruption in gut microbiota is thought to contribute to the development of depression-like behaviors. Interestingly, dietary supplementation with prebiotics has been shown to increase *Lactobacillus* abundance, helping to alleviate these behaviors in LH mice (59). Additionally, compared to LH-resilient rats, LH-susceptible rats exhibit a notable increase in certain bacterial genera in the gut, including *Asaccharobacter*, *Eisenbergiella*, and *Klebsiella* (60). Alongside the LH model, other methods such as drug treatments and surgical interventions are also used to induce depression in rodents. For example, corticosterone (CORT) is commonly administered to mice to induce depression, which results in a decrease in microbial diversity and an increase in *Lactobacillus vaginalis* and unclassified *Bifidobacterium* species (61). A study by Jiang et al. underscores the importance of gut microbiota in reducing anxiety and depression-like behaviors in post-stroke mice (62).

In addition to rodents, non-human primates are also used to establish depression models due to their close similarities to humans in terms of physiology, cognitive abilities, neuroanatomy, social complexity, reproduction, and development. In female cynomolgus monkeys, which naturally display depression-like behaviors, characteristic dysbiosis of the Firmicutes phylum has been observed (63). When healthy male cynomolgus monkeys of varying ages are exposed to CUMS, significant differences in microbial composition and gut-brain metabolic characteristics emerge between adolescent and adult monkeys. Dysbiosis of *Clostridium* and *Haemophilus* is observed only in adolescent depressed monkeys, while it is absent in adults. These findings strongly suggest that gut microbial dysbiosis is not just a consequence of depression but also plays a critical role in its development. The connection between gut microbiota and mood disorders underscores the potential of microbiome-based therapeutic strategies aimed at modulating the gut-brain axis to alleviate depression-like behaviors. Overall, these cross-species studies emphasize the pivotal role of the gut microbiome in influencing neuropsychiatric outcomes, making it a promising target for future therapeutic approaches.

### Clinical studies

As shown in **Table 2**, numerous clinical studies have highlighted significant differences in the gut microbiota composition between individuals with MDD and healthy controls, emphasizing the critical role of the MGBA in the pathophysiology of depression (28, 66–69). In healthy individuals, the gut microbiome is predominantly composed of Firmicutes (79%), followed by Bacteroidetes (17%), Actinobacteria (3%), Proteobacteria (0.9%), and Verrucomicrobia (0.1%) (70). However, notable alterations in microbiota have been reported in MDD patients, with increased abundance of Enterobacteriaceae and *Alistipes*, alongside a reduction in *Faecalibacterium* levels, which correlate with the severity of depressive symptoms (71). Similarly, female MDD patients exhibit an enrichment of Bacteroidetes, Proteobacteria, and Fusobacteria, while healthy controls show higher levels of Firmicutes and Actinobacteria (72). Subsequent meta-analyses have confirmed these findings, showing consistently reduced levels of *Coprococcus* and *Faecalibacterium* in MDD patients (73). Furthermore, probiotic interventions have been shown to significantly alleviate depressive symptoms, supporting the therapeutic potential of microbiota modulation in treating depression (74). Importantly, changes in the gut microbiota of MDD patients are considered closely related to their somatic symptoms (75). Notably, antidepressant treatment can induce dynamic changes in the gut microbiota. In a longitudinal study by Wang et al., untargeted metabolomics and metagenomic sequencing were performed on blood and fecal samples collected from 110 MDD patients at three timepoints (baseline, week 2, and week 12) during escitalopram (ESC) treatment (76). The findings showed that while the gut microbial composition did not change significantly by week 2, notable alterations emerged by week 12, with the most pronounced changes observed in spore-forming bacteria.

**Table 2:** Changes in gut microbiota of patients with MDD in clinical studies.

Group	Method	Result	Ref
MDD patients (n=46; 29 active-MDD and 17 responded-MDD) HC (n=30)	Pyrosequencing was used to analyze the fecal samples of patients.	• Enterobacteriaceae and *Alistipes*↑, Firmicutes↓ • Negative correlation was observed between *Faecalibacterium* and the severity of depressive symptoms	(71)
MDD patients (n=160) HC (n=101)	16S rRNA gene sequencing was employed to analyze the composition of fecal microbiota ELISA was used to measure inflammatory cytokines	• *Bifidobacterium*, *Blautia*, *Haemophilus* ↑ • *Bacteroides*, *Faecalibacterium*, *Roseburia*↓ • *Bacteroides* and *Roseburia* negatively correlated with the hs-CRP, HAMD-24, the total and factor scores of SSS in all participants	(75)
MDD patients with ESC treatment (n=110) HC (n=166)	Metabolomics analysis and metagenomic sequencing were conducted to examine the blood and fecal samples of two groups of individuals at different time points.	• The use of ESC leads to a decrease in microbial abundance and function • Significant changes occurred in the gut microbiota after 12 weeks of treatment, with the most significant change observed in spore forming bacteria	(76)
MDD patients (n=70; 25 young and 45 middle-aged MDD) HC (n=71; 27 young and 44 middle-aged HC)	Detection of feces using 16S rRNA gene sequencing	• Firmicutes↓ and Bacteroidetes↑ compared with young HC • Bacteroidetes↓ and Actinobacteria↑ compared with middle-aged HCs • Nine bacterial taxa at the genus level differ between young and middle-aged MDD patients	(81)
MDD patients (n=44; 24 female and 20 male MDD) HC (n=44; 24 female and 20 male HC)	Detection of feces using 16s rRNA gene sequencing	• Bacteroides was reduced in male MDD patients, while Actinobacteria was elevated in females	(82)
PPD patients (n=39) HC (n=18)	Serum sex hormone levels were measured by ELISA Fecal samples were collected for 16S rRNA gene sequencing	• *Faecalibacterium*, *Phascolarctobacterium*, *Butyricicoccus*, and Lachnospiraceae↓, Enterobacteriaceae family↑in PPD patients • Lachnospiraceae and *Faecalibacterium* were linked to sex hormone levels	(89)
Patients with absent or mild depressive symptoms (n=16) Patients with moderate or severe depressive symptoms (n=18)	Fecal samples were collected at late pregnancy and postpartum for 16S rRNA gene sequencing, and SCFAs were quantified using GC-MS.	• Postpartum women with moderate to severe symptoms showed a significant increase in Enterobacteriaceae abundance • No differences in SCFA concentrations were found between the two groups	(90)
MDD patients (n=49) Probiotic group (n = 24) Placebo group (n = 25)	Participants’ scores on the HAMD-17, IDS, HAMA, and GAD-7 were assessed at various time intervals.	• Probiotics can significantly improve depressive and anxiety symptoms in MDD patients	(92)

(The ↑/↓ are shown as P<0.05).

ELISA, Enzyme-linked immunosorbent assay; GAD-7, Generalized Anxiety Disorder-7; GC-MS, Gas chromatography-mass spectrometry; HAMA, Hamilton Anxiety Scale; HAMD, Hamilton Depression Scale Untargeted; HC, Healthy control; hs-CRP, High-sensitivity C-reactive protein; IDS, Inventory of Depressive Symptomatology; MDD, Major depressive disorder; PPD, Postpartum depressive disorder; SCFAs, Short-chain fatty acids; SSS, Somatic Self-rating Scale.

In addition to shifts in microbial composition, the dynamic evolution of the gut microbiota throughout the lifespan further complicates the relationship between microbiota and depression (77–80). Emerging evidence suggests that MDD patients across various age groups may display distinct microbial profiles, highlighting the importance of age-specific approaches in microbiota-based therapies (81). Additionally, differences in gut microbiota composition have been observed between male and female MDD patients. Notably, the level of Bacteroides was significantly reduced only in male MDD patients, while the level of Actinobacteria was significantly elevated only in female MDD patients (82). Alterations in the gut microbiota have also been linked to specific physiological periods, such as pregnancy, which can further impact the onset and progression of depression (83–85). Postpartum depression (PPD), a common subtype of MDD affecting around 10%-15% of women (86), not only negatively affects maternal health and mother-infant bonding but also carries long-term implications for child development (87, 88). Recent studies have revealed significant differences in microbial diversity and composition between PPD patients and healthy postpartum women. For instance, Zhou et al. found partial differences in microbial diversity between PPD patients and controls, with certain bacterial taxa, such as Lachnospiraceae and *Faecalibacterium*, correlating with fluctuations in sex hormone levels (89). Additionally, an increased abundance of Enterobacteriaceae has been observed in postpartum women experiencing severe depressive symptoms (90).

More strikingly, several clinical trials have validated the effectiveness of probiotic supplementation in alleviating depressive symptoms by modulating the gut microbiota (29, 91). One randomized controlled trial (RCT) reported significant reductions in depression scale scores following probiotic supplementation (92). Additionally, numerous studies have confirmed that specific probiotic strains can significantly improve maternal mood, further supporting the therapeutic benefits of microbiota-based interventions (93). Furthermore, studies have shown that supplementation with the right probiotics can effectively enhance maternal mood (93). Meanwhile, probiotics have been found to improve verbal episodic memory and increase serum levels of brain-derived neurotrophic factor (BDNF) in MDD patients (25). In parallel, microbiota-based biomarkers are increasingly being utilized to differentiate between depressed individuals and healthy controls. Recent advancements in genomic sequencing, such as single-nucleotide-resolved amplicon sequence variants (ASVs) of human gut microbiomes, have allowed the identification of depression phenotypes in healthy cohorts, emphasizing the value of gut microbiota profiling in clinical diagnostics (94). These findings not only highlight the clinical significance of microbiota dysbiosis as a potential biomarker for depression but also underscore the promising therapeutic potential of modulating the microbiota-gut-brain axis in psychiatric treatments.

## MGBA in depression pathogenesis

**Figure 1:**
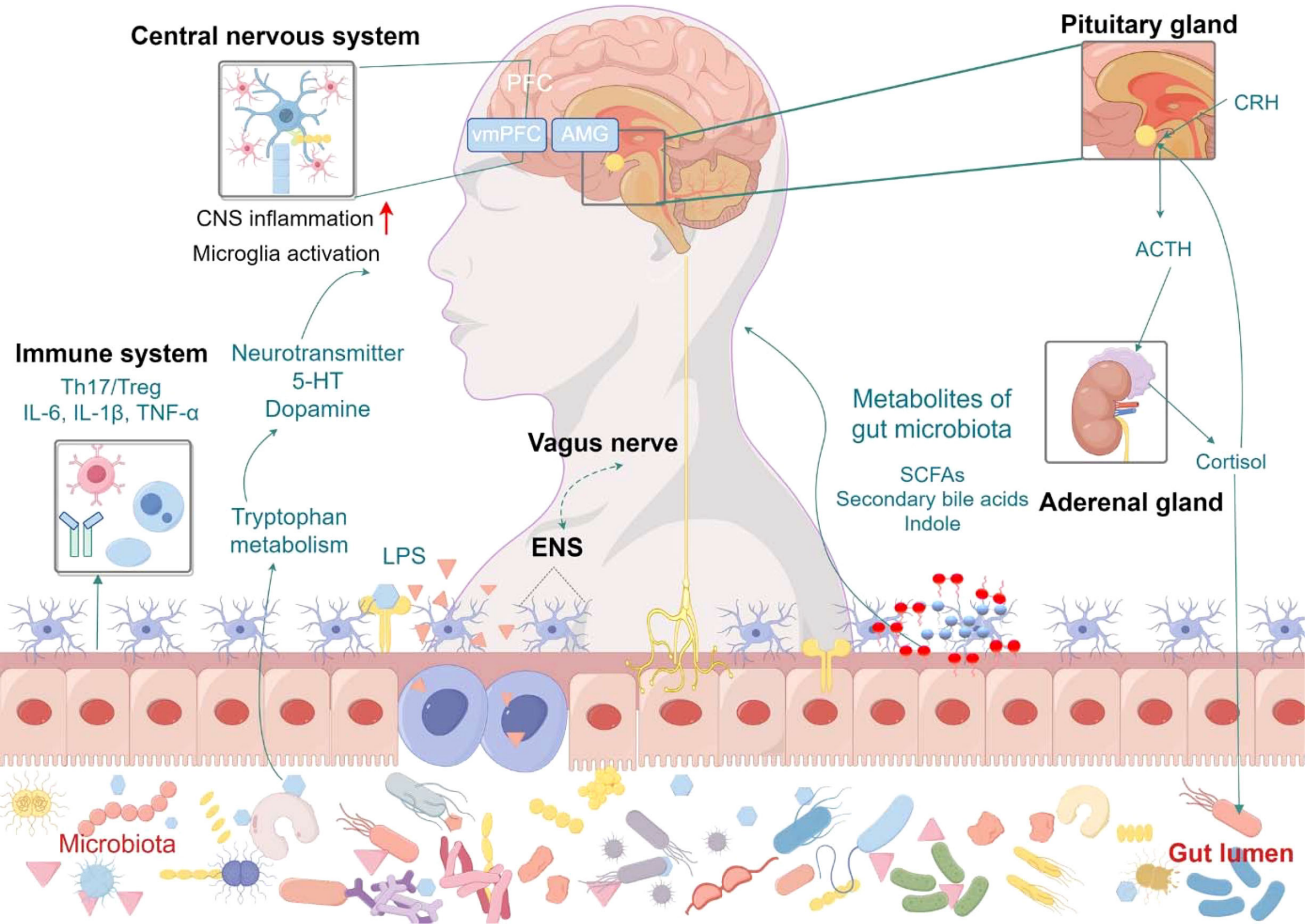
Mechanisms of gut microbiota in depression. The gut microbiota influences depression through three interconnected pathways: neural signaling, endocrine regulation, and immune modulation. Vagus nerve-mediated bidirectional communication allows gut-derived neurotransmitters (e.g., serotonin, GABA) to directly affect central nervous system activity, while activation of the hypothalamic-pituitary-adrenal (HPA) axis by microbial metabolites (e.g., short-chain fatty acids, bile acids) induces cortisol release, promoting neuroinflammation and synaptic dysfunction in brain regions like the hippocampus. Intestinal barrier dysfunction permits bacterial components and proinflammatory cytokines (e.g., IL-6, TNF-α) to translocate systemically, activating microglia and disrupting Th17/Treg cell balance in both gut and brain, thereby exacerbating depressive symptomatology. ACTH, Adrenocorticotropic Hormone; AMG, Amygdala; CNS, Central Nervous System; CRH, Corticotropin Releasing Hormone; ENS, Enteric Nervous System; 5-HT, 5-hydroxytryptamine; LPS, Lipopolysaccharide; SCFAs, Short-chain fatty acids; vmPFC, Ventromedial Prefrontal Cortex.

The relationship between gut microbiota and depression has garnered increasing attention in recent years, as research efforts aim to unravel the complex mechanisms that link these two entities and identify potential therapeutic avenues. The gut-brain axis, a bidirectional communication network between the gastrointestinal tract and the central nervous system, is integral to maintaining physiological homeostasis. Gut microbiota plays a central role in this axis, influencing brain function through multiple signaling pathways. As such, the MGBA hypothesis has gained prominence as a potential framework for understanding the pathophysiology of depression. Current research suggests that gut microbiota influence the onset and progression of depressive disorders through three primary mechanisms: neural signaling, endocrine modulation, and immune regulation (**Figure 1**). These pathways contribute to altering brain function and mood regulation, providing new insights into the therapeutic potential of targeting the gut microbiota in treating depression.

### Neural signaling

In the MGBA, the neural signaling network plays a crucial role in facilitating rapid communication between the gut and the brain. This network integrates the enteric nervous system (ENS), the vagus nerve, and spinal nerves, each of which contributes to the regulation of gut-brain signaling pathways and the pathophysiology of neuropsychiatric disorders, including depression (95). The ENS, an intricate and expansive neural network embedded within the gastrointestinal tract, not only regulates the gut environment but also shares neurotransmitters with the CNS. Importantly, the ENS has been implicated in depression-related alterations, contributing to the development of mood disorders (96). In addition to its regulatory role in intestinal functions, the ENS modulates gut microbial composition, influences microbiota-derived metabolites, adjusts neurotransmitter levels, and participates in immune signaling (10, 97, 98). Moreover, the ENS is subject to modulation by the gut microbiota, with its development, function, and renewal being heavily influenced by microbial interactions (99). Pathological disruptions in ENS function can exacerbate the course of depression by disturbing key processes such as gut secretion, immune responses, and intestinal barrier integrity (100).

The vagus nerve is another critical component of the MGBA, especially in the context of depression. It represents one of the most direct pathways for microbial signals to reach the brain (101, 102). Notably, early work by Bravo et al. (103) demonstrated that oral administration of *Lactobacillus rhamnosus* alleviated stress-induced depressive-like behaviors in mice, with this effect being blocked following vagotomy, underscoring the vagus nerve’s central role in microbial-mediated signaling (103). Subsequent studies have provided further insights into the vagus nerve’s involvement in depression. Zhang’s group found that lipopolysaccharide (LPS)-induced depressive-like behaviors in mice were entirely abolished following subdiaphragmatic vagotomy (SDV) (104). This work revealed that LPS administration altered gut microbiota composition, with a notable increase in the abundance of Firmicutes and Bacteroidetes, changes that were not observed in SDV mice, suggesting the critical role of the vagus nerve in mediating microbial-induced alterations in the gut. Moreover, the same research demonstrated that continuous administration of *Lactobacillus intestinalis* and *Lactobacillus reuteri* for 14 days induced behavioral despair, accompanied by elevated plasma IL-6 levels and reduced prefrontal cortex synaptic protein expression, effects which were effectively blocked by SDV (105). In a RCT, Mörkl et al. (106) observed significant improvements in vagal nerve function following probiotic treatment in patients with MDD, which correlated with increased abundances of Christensenellales and *Akkermansia muciniphila* (106). Together, these studies emphasize the pivotal role of the vagus nerve in mediating the gut-brain communication in depression.

Neurotransmitters serve as fundamental mediators of neural communication, and their dysregulation is a key factor in the pathogenesis of depression. Most contemporary antidepressant therapies target the modulation of synaptic neurotransmitter concentrations (107). Among these, serotonin (5-HT) deficiency is strongly linked to mood disorders (108), with gut microbiota exerting a significant influence on 5-HT levels, and gut microbiota can influence emotional states by regulating 5-HT levels. Selective serotonin reuptake inhibitors (SSRIs), which act on this system, have become widely used for treating mood disorders (109, 110). Notably, approximately 95% of the body’s 5-HT is synthesized in the gut (111), highlighting the critical role of the intestinal microbiota in regulating emotional states by modulating 5-HT production. In seminal work by William et al., germ-free (GF) mice exhibited a 2.8-fold reduction in 5-HT levels compared to conventionally raised controls, suggesting the essential role of the microbiota in regulating serotonin levels (112). Moreover, Zhou et al. demonstrated that FMT from healthy adolescent volunteers significantly elevated 5-HT concentrations in the brain and colon of adolescent mice subjected to chronic restraint stress (CRS), indicating that the microbiota directly influences CNS neurotransmitter levels (113). Several studies have also demonstrated the antidepressant effects of probiotics such as *Bifidobacterium* (114, 115) and *Lactobacillus* (116) strains through the modulation of 5-HT levels, offering potential therapeutic avenues for mood disorders. Additionally, oral administration of 5-HT not only alleviated depression-like behaviors in mice but also reversed depression-induced alterations in SCFAs concentrations and brain-derived neurotrophic factor (BDNF) levels, while restoring gut microbiota balance (117).

Beyond serotonin, the gut microbiota also influences the synthesis of other neurotransmitters such as gamma-aminobutyric acid (GABA), dopamine, and acetylcholine, all of which are integral to emotional regulation. The genus *Bacteroides*, for instance, encodes glutamate decarboxylase (GAD), a key enzyme involved in GABA synthesis (118). Both *Bifidobacterium* and *Lactobacillus* strains have also been shown to synthesize GABA (119), with the administration of GABA-producing strains leading to depression-like behaviors in animal models (120). Furthermore, research suggests that chronic stress can reduce the abundance of urease-positive bacteria in the gut, leading to lower peripheral ammonia levels. This disruption in ammonia homeostasis decreases glutamine synthesis in astrocytes, which in turn promotes GABAergic dysfunction (121). Furthermore, the gut microbiota significantly influences dopamine metabolism, a neurotransmitter central to reward processing and mood regulation (122). For instance, *Enterococcus faecalis* has been shown to alleviate depressive symptoms in mice through dopaminergic pathways (123). In a randomized, double-blind, placebo-controlled clinical trial, treatment with *Bifidobacterium breve* BB05 reduced fecal levels of acetylcholine (ACh), epinephrine (Epi), and norepinephrine (NE), which was accompanied by improvements in both anxiety and depressive symptoms (124). Thus, the gut microbiota profoundly impacts the neural signaling pathways within MGBA, modulating neurotransmitter synthesis and influencing brain-gut communication, ultimately affecting the pathophysiology of depression.

### Endocrine modulation

The HPA axis is a central neuroendocrine system (125) that plays a crucial role in regulating the body’s response to stress, and it is intimately linked with the MGBA through bidirectional interactions (126, 127). Dysregulation of the HPA axis is frequently observed in individuals with depression, characterized by elevated secretion of corticotropin-releasing hormone (CRH) and adrenocorticotropic hormone (ACTH), increased plasma cortisol concentrations, and a disrupted negative feedback mechanism (128). These alterations in HPA axis function are believed to contribute to gastrointestinal inflammation, compromised gut barrier integrity, and neuronal damage (129–132), ultimately resulting in shifts in the gut microbiome composition. Conversely, gut microbiota can exert significant influence on HPA axis function. Dysbiosis has been shown to facilitate the translocation of pro-inflammatory cytokines such as interleukin-1β (IL-1β), IL-6, and tumor necrosis factor-α (TNF-α) across the blood-brain barrier, which in turn activates the HPA axis, intensifying the stress response (30). Notably, the GABA signaling pathway plays a critical role in the negative feedback regulation of the HPA axis. It modulates the axis’s responsiveness to stress, potentially alleviating HPA axis dysfunction (133). Numerous studies have demonstrated that the gut microbiota can influence GABA metabolism, further emphasizing its role in the regulation of HPA axis activity (118, 134). For instance, Li et al. reported that *Bifidobacterium breve* 207–1 modulates GABA and related hormones in the context of the gut-brain axis, suggesting a potential mechanistic link between microbiota and HPA axis regulation (135).

In addition to GABA signaling, the gut microbiota can also affect enteroendocrine cells via microbial metabolites like SCFAs and secondary bile acids. SCFAs, which are produced through bacterial fermentation of partially indigestible polysaccharides such as dietary fiber and resistant starch, are among the most crucial microbial metabolites (136). The primary SCFAs—acetate, propionate, and butyrate—constitute over 95% of total SCFA production, with other metabolites, such as lactic acid, present in smaller amounts (137). These SCFAs exert a variety of physiological effects within the gut, including regulation of redox balance, maintenance of intestinal pH homeostasis, promotion of hormone secretion, and involvement in epigenetic modifications (138–141). Recent research has spotlighted the significant role of SCFAs in neuropsychiatric disorders, particularly depression. Studies have demonstrated that chronic stress leads to a marked reduction in SCFA levels—such as acetate, propionate, and valerate—in animal models. Moreover, a positive correlation has been observed between the abundance of *Allobaculum* and acetic acid levels in these models (142). Similarly, in post-stroke depression models, decreased levels of butyrate, acetate, and valerate are linked with alterations in lipid metabolism (143). Notably, the depletion of butyrate, a metabolite known for its anti-inflammatory effects, has been directly associated with the manifestation of depressive symptoms (144). Additionally, research on rats exposed to blue light during sleep revealed increased lactic acid levels in the cerebrospinal fluid and lateral habenula, correlating with the onset of depressive behaviors. Importantly, inhibiting lactic acid production in these rats alleviated these depressive-like symptoms (145). SCFAs may contribute to the pathophysiology of depression through several mechanisms, including immunomodulation of Th17/Treg cells (146), activation of the TLR4/NF-κB inflammatory pathway (147), regulation of acetyl-CoA synthetase short-chain family member 2 (ACSS2) (148), and histone epigenetic regulation (149). Emerging evidence suggests that SCFAs play a crucial role in maintaining blood-brain barrier (BBB) integrity (150, 151), while BBB impairment has been implicated in depression pathogenesis (152, 153). These findings underscore the pivotal role of SCFAs in modulating the MGBA, suggesting that strategies aimed at modulating SCFA production or signaling could hold therapeutic potential for treating depression.

Furthermore, the gut microbiota significantly influences bile acid metabolism, with specific bacteria, such as *Clostridium* and *Bacteroides*, being capable of synthesizing secondary bile acids (96). Clinical and animal studies have revealed a compelling relationship between bile acids and depressive disorders. In a comparison of serum and fecal samples from individuals with MDD and healthy controls, researchers found significantly elevated levels of 2,3-deoxycholic acid in MDD patients, while taurolithocholic acid (TLCA), glycolithocholic acid (GLCA), and 3-sulfolithocholic acid were found at lower concentrations (154). Notably, a positive correlation was observed between the abundance of *Verrucomicrobium* and the levels of TLCA and GLCA, further illustrating the microbiota-bile acid axis in depression. Similarly, increased dissociation of conjugated bile acids and enhanced biosynthesis of secondary bile acids have been observed in CUMS mice, with an increased abundance of Ruminococacae promoting the biosynthesis of deoxycholic acid (DCA) (155). These findings collectively highlight the intricate role of bile acid metabolism in the pathophysiology of depression and suggest that modulation of the bile acid microbiota could represent a novel avenue for therapeutic intervention.

Gut microbiota plays a crucial role in the pathogenesis of depression through the metabolism of tryptophan into indole and its derivatives. Gut microbes, equipped with a variety of catalytic enzymes, transform tryptophan into several indole metabolites, including indole-3-propionic acid (IPA), indole-3-acetaldehyde (IAld), indole-3-acetic acid (IAA), and indole-3-lactic acid (ILA) (156). A growing body of evidence has linked these indole metabolites to depression-related behavioral changes. For example, Brydges et al. found that serum concentrations of indoxyl sulfate (IS), an indole metabolite, are positively correlated with the severity of depressive and anxiety symptoms (157). Similarly, a prospective observational study revealed significantly elevated urinary IS concentrations in women with recurrent depressive episodes (158). Other indole derivatives have also been implicated in depression’s pathophysiology. In one study, Qian et al. showed that hippocampal ILA levels were significantly reduced in a mouse model of depression. Supplementation with Bifidobacteria not only increased ILA concentrations but also alleviated neuroinflammation and improved depression-related phenotypes by activating the aryl hydrocarbon receptor (AhR) signaling pathway (159). Furthermore, other studies have shown that reduced IAld levels are associated with worsened depressive symptoms in obese patients (160). In animal models, indole-3-carboxaldehyde (I3C) was found to mediate depressive behaviors induced by chronic restraint stress. Both I3C supplementation and administration of *Lactobacillus reuteri*, an I3C-producing strain, were effective in ameliorating these behavioral deficits (161).

### Immune regulation

Recent studies have increasingly highlighted the connection between immune system activation and the onset of depression, suggesting that inflammation plays a crucial role in its pathophysiology (159, 162–164). Key pro-inflammatory cytokines implicated in depression include IL-6, TNF-α, IL-1β, IL-10, IL-1ra, transforming growth factor-β, and C-reactive protein (CRP) (165, 166). The regulation of these cytokines occurs through a complex immune signaling network that integrates both innate and adaptive immune responses in the gut, brain, and systemic circulation, thereby facilitating the interaction between immune functions, gut microbiota, and depression (167–169).

One of the central mechanisms through which inflammation influences depression is altered intestinal permeability, commonly referred to as leaky gut syndrome (LGS) (170). LGS results from dysbiosis of the gut microbiota, epithelial damage, and compromised intestinal barrier function, which collectively contribute to neuroinflammation—a critical driver in the pathogenesis of depression (171). Furthermore, the compromised gut barrier allows for the translocation of LPS-producing Gram-negative bacteria, triggering immune responses that exacerbate depression (172). The role of LPS in MDD has been well-established (173), with LPS-induced inflammation models serving as valuable tools for investigating the mechanisms underlying MDD (174–176). Targeting the regulation of gut microbiota has emerged as a promising strategy for mitigating LPS-induced depressive symptoms. For example, *Limosilactobacillus fermentum* L18 has been shown to restore intestinal epithelial permeability by enhancing the expression of tight junction proteins such as occludin and E-cadherin, which can improve gut-barrier integrity (177). Similarly, Ramalho et al. demonstrated that administering *Lactococcus lactis* in LPS-induced depressive mice improved related behaviors by modulating oxidative stress and pro-inflammatory cytokine levels in the hippocampus (178).

The concept of the gut microbiota-immune-glia axis has also been introduced, offering a deeper understanding of the bidirectional communication between the gut microbiota and glial cells in the brain (169). Glial cells, including microglia (179, 180), astrocytes (181), oligodendrocytes (182), and ependymal cells (183), have been shown to play significant roles in the development of depression. The gut microbiota influences the activation and function of these glial cells through neural and chemical signaling pathways. In particular, microglia exhibit dynamic shifts between pro-inflammatory and anti-inflammatory states, and dysregulation of this process is thought to contribute to the neuroinflammation observed in depression (184, 185). For instance, rifaximin, a gut microbiota-targeted treatment, has been found to alleviate depressive behaviors in CUMS mice. This effect correlates with shifts in the gut microbiota, particularly the relative abundance of Ruminococcaceae and Lachnospiraceae, which subsequently influence brain microglia and peripheral cytokine levels (such as TNF-α, IL-1β, IL-10) (186). Similarly, minocycline has demonstrated antidepressant effects via its modulation of the gut microbiota-microglia regulatory pathway (187). Collectively, these findings suggest that targeting microglial activation through modulation of the gut-brain axis may represent a promising therapeutic strategy for depression (188, 189).

Immune cells, particularly macrophages, also play critical roles in the pathophysiology of depression. Macrophages are central to innate immune responses and activate adaptive immunity, including the differentiation of T lymphocytes into pro-inflammatory and anti-inflammatory subsets (190). Within the context of depression, the balance between Th17 and Treg cells—two distinct T cell subsets—has gained attention. Th17 and Treg cells, which are influenced by the gut microbiota (191, 192), have been implicated in regulating brain development, neuroinflammation, and the activation of glial cells such as microglia and astrocytes during periods of stress (184, 193, 194). Increasing evidence suggests that an imbalance between Th17 and Treg cells plays a role in depression (195–197). The dysregulated ratio of Th17 to Treg cells may contribute to the chronic inflammation observed in depressed individuals, and the restoration of this balance could have therapeutic benefits. Research by Westfall et al. revealed that metabolites from the gut microbiota can modulate the Th17/Treg ratio, alleviating stress-induced inflammatory responses and improving resilience against anxiety and depressive behaviors (146). Notably, this gut microbiota-mediated regulation of the Th17/Treg imbalance is also thought to influence the efficacy of antidepressant treatments, such as ketamine (198–200). Thus, modulating immune responses and restoring balance within the immune system and gut microbiota may provide valuable insights into novel approaches for treating depression.

## Therapeutic potential of gut microbiota in depression treatment

**Figure 2:**
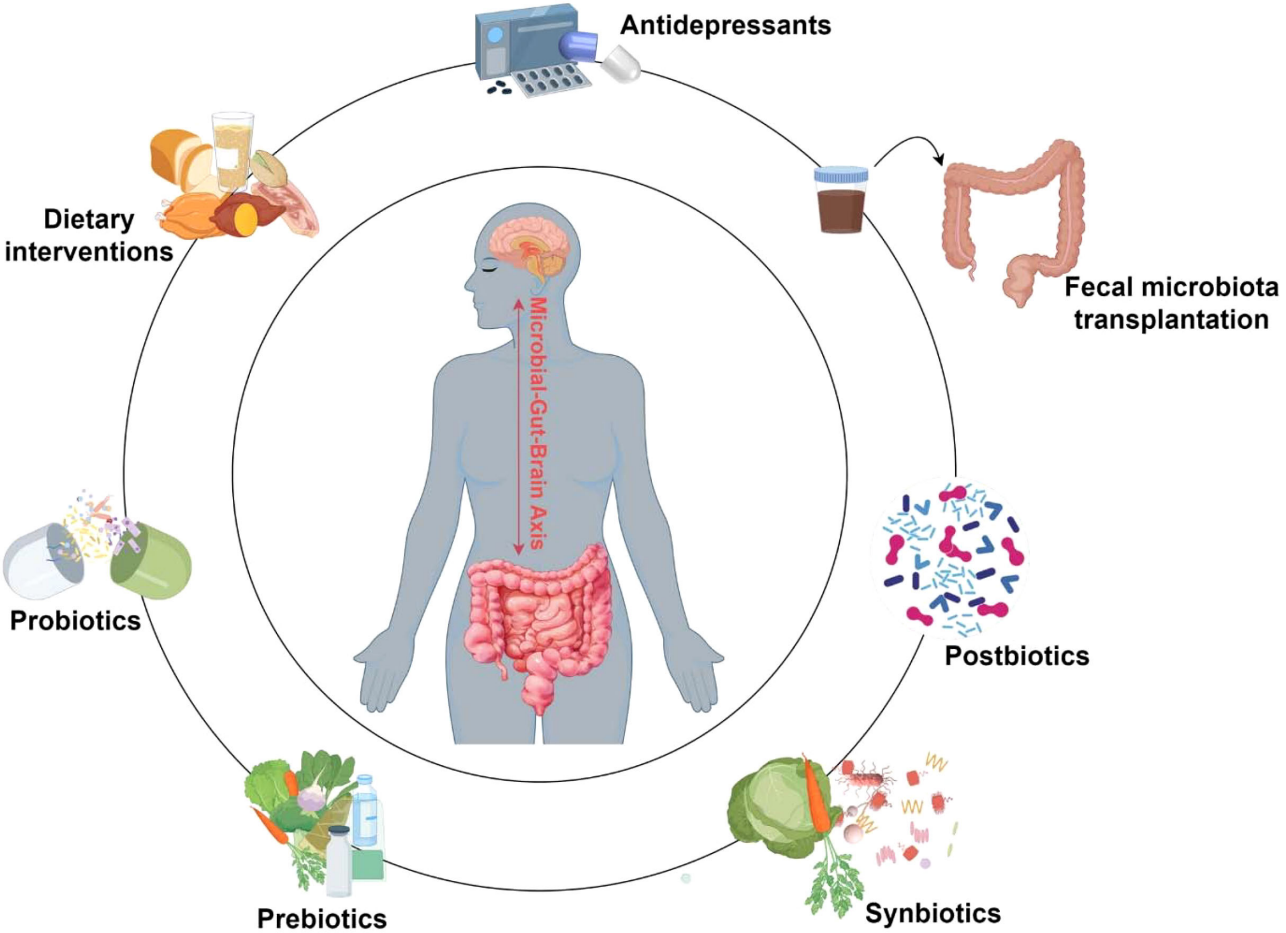
Applications of gut microbiota in depression treatment. This diagram summarizes emerging gut microbiota modulation strategies for depression treatment. Antidepressants, dietary interventions, and microbiota-based therapies (including probiotics, prebiotics, synbiotics, postbiotics, and fecal microbiota transplantation) can influence gut microbial composition and function, potentially alleviating depressive symptoms. These strategies—used individually or synergistically—have demonstrated substantial promise as adjunctive therapies for depression, offering novel pathways to enhance treatment efficacy.

MGBA axis plays a pivotal role in the regulation of neurological function and mental health, with growing evidence supporting its involvement in the pathophysiology and treatment of depression. Unlike the static nature of human genetics, the gut microbiota is a highly dynamic, evolving, and diverse ecosystem that is responsive to various external influences. This plasticity presents an opportunity for therapeutic intervention aimed at restoring balance within the microbiota to improve mental health outcomes. Recent research has highlighted the potential of modulating the gut microbiota through various approaches, such as dietary interventions, administration of probiotics, prebiotics, synbiotics, and postbiotics, and the use of FMT. These strategies can promote beneficial shifts in the gut microbiome, which, in turn, can positively influence the gut-brain signaling pathways involved in depression. By combining these microbiota-modulating strategies with traditional antidepressant therapies, there is significant promise in enhancing the efficacy of depression treatment through a more comprehensive approach that targets both the microbiome and the CNS. The emerging therapeutic role of the gut microbiota in depression underscores the importance of the gut-brain axis as a critical modulator of mental health and provides a promising avenue for the development of novel treatment strategies (**Figure 2**).

### Interaction between gut microbiota and antidepressants

Pharmacological intervention remains the cornerstone of treatment for depression, with oral antidepressants being the most prescribed modality. These pharmacological agents can have both direct and indirect effects on the gut microbiota, influencing its composition and function (201, 202). Antidepressants, such as selective serotonin reuptake inhibitors (SSRIs), tricyclic antidepressants (TCAs), and selective serotonin and norepinephrine reuptake inhibitors (SNRIs), have been shown to alter microbial diversity and composition in various ways (203–207). For instance, SSRIs have been reported to increase the abundance of *Bacteroides*, while TCAs are associated with elevated levels of *Clostridium* in the gastrointestinal tract (208). Additionally, norepinephrine reuptake inhibitors, such as reboxetine (RBX), have been found to significantly reduce *Lactobacillus* populations and decrease the Firmicutes/Bacteroidetes ratio, both of which are key indicators of microbial dysbiosis (209). These alterations in gut microbiota may contribute to the gastrointestinal side effects often observed with antidepressant treatment, such as nausea, bloating, and altered bowel movements (210).

Conversely, the gut microbiota can influence the pharmacokinetics and pharmacodynamics of antidepressants. Gut bacteria may modulate the metabolism of these drugs through enzymatic mechanisms, which can impact both their therapeutic efficacy and toxicity (211, 212). For example, Klünemann et al. demonstrated that intestinal bacteria, such as *Streptococcus salivarius* and *Escherichia coli* IAI1, can enhance the bioaccumulation of the SNRI venlafaxine, potentially reducing its bioavailability and therapeutic effectiveness (213). This highlights a crucial interaction in which the microbiota could diminish the expected clinical response to antidepressants. Furthermore, treatment resistance in MDD has been linked to specific gut microbial profiles (214). Patients who exhibit resistance to standard antidepressant treatments often have elevated levels of Proteobacteria, Tenericutes, and Peptostreptococcaceae, while the presence of *Thaumarchaeota*, *Yersinia*, and *Peptococcus* may be indicative of treatment failure (215). In the context of novel therapeutic approaches, ketamine has emerged as a promising alternative due to its higher efficacy and lower side-effect profile. Recent studies suggest that the gut microbiota plays a role in potentiating ketamine’s antidepressant effects, with specific taxa, such as Actinobacteria and Coriobacteria, being identified as facilitators of its therapeutic action (216). Despite its potential, antidepressant treatment, especially in the context of MDD, often comes with a spectrum of adverse effects, including weight gain, gastrointestinal disturbances, hormonal imbalances, blurred vision, and an increased risk of cardiovascular events (210, 217–220). Probiotics, as an adjunctive treatment, have shown promise in alleviating some of these side effects, further emphasizing the potential of microbiota-targeted therapies in depression management (221).

It is important to note that the current body of research primarily focuses on single drug-microbe interactions. However, in clinical settings, many patients with MDD are prescribed a combination of medications, which may lead to more complex interactions with the gut microbiome. This polypharmacy approach could result in a more intricate regulatory network within the microbiota, potentially amplifying or mitigating the effects of individual drugs. Although the precise mechanisms underlying these interactions are still not fully understood, preliminary evidence supports the potential of microbiota-modulated antidepressant therapies. As our understanding of these complex interactions evolves, microbiome-based adjunctive treatments are expected to become an increasingly integral component of depression therapy.

### Dietary interventions in gut microbiota-depression interactions

The composition and functionality of the gut microbiota are highly responsive to dietary influences, which, in turn, can significantly impact mental health outcomes, including depression (222). A growing body of evidence supports the notion that dietary interventions can play a crucial role in preventing and managing depression by modulating the gut microbiota. Research has consistently shown that healthy dietary patterns can enhance mental well-being, while poor dietary choices can exacerbate depressive symptoms (223). The Western diet, which is emblematic of modern eating habits, is typically characterized by high consumption of ultra-processed foods, red meat, refined sugars, and trans fats, with a concomitant deficiency in fruits, vegetables, and whole grains (224). This dietary pattern is associated with several detrimental effects on gut microbiota, including a reduction in microbial diversity, an increase in the abundance of pathogenic bacteria, and the promotion of systemic inflammation, all of which are linked to an elevated risk of depression (225, 226). Specifically, an imbalance in gut microbial populations caused by the Western diet may trigger neuroinflammatory pathways that contribute to mood disorders. In contrast, the Mediterranean diet has garnered significant attention for its protective effects against depression (227). This diet, rich in fruits, vegetables, whole grains, legumes, nuts, and olive oil, with moderate intake of fish and poultry, has been shown to reduce the incidence and severity of depressive symptoms (228). The Mediterranean diet promotes a favorable gut microbiota composition by decreasing the prevalence of pathogenic bacteria such as *Escherichia coli* and increasing the abundance of beneficial species, including *Bifidobacterium*. These microbial shifts lead to enhanced production of SCFAs, particularly butyrate, which has been identified as a key player in mediating the positive effects of this diet on gut and brain health (229). SCFAs have been shown to reduce systemic inflammation and neuroinflammation, thereby lowering the risk of depression (230, 231).

Additionally, diets from countries such as Norway and Japan, which are rich in omega-3 polyunsaturated fatty acids, whole grains, olive oil, soy products, and low-fat dairy, have been associated with a decreased risk of depression (232–234). The inclusion of ω-3 fatty acids, in particular, has been linked to beneficial changes in gut microbiota composition and a reduction in neuroinflammation, further supporting the idea that dietary patterns can modulate both gut health and mental well-being. The rising popularity of vegetarian diets has also spurred interest in their relationship with depression. Vegetarianism, which emphasizes plant-based foods such as vegetables, whole grains, legumes, nuts, seeds, and fruits (235), has been suggested in some studies to offer protective effects against depression (236). However, findings on the relationship between vegetarianism and depression are mixed, with some studies reporting a reduced risk of depressive symptoms, while others suggest no significant effects or even potential risks due to nutrient deficiencies (237, 238). These discrepancies underscore the need for more research to elucidate the mechanisms underlying the effects of vegetarian diets on mental health. The ketogenic diet, characterized by high fat, moderate protein, and very low carbohydrate intake, is another dietary intervention that has attracted attention for its potential antidepressant effects. Through its effects on neurotransmitter systems, particularly glutamate and GABA transmission, as well as its ability to modulate oxidative stress and inflammation, the ketogenic diet has shown promise in treating depression (239–241). However, the long-term sustainability of the ketogenic diet remains a concern due to its potential to cause nutritional imbalances, particularly with respect to carbohydrates and micronutrients, which could lead to metabolic disruptions if followed over extended periods.

Taken together, there is substantial evidence supporting the role of dietary patterns in shaping the gut microbiota and influencing mental health, particularly in relation to depression. While various diets, such as the Mediterranean, Norwegian, Japanese, and vegetarian diets, have demonstrated benefits in modulating depression risk, further studies are needed to clarify the underlying mechanisms and optimize dietary interventions as adjunctive treatments for mood disorders.

### Microecologics in adjunctive depression therapy

Microecologics encompass viable bacteria, non-viable bacteria, and their metabolites, including probiotics, prebiotics, synbiotics, and postbiotics. These agents have garnered attention for their ability to modulate the gut microbiota, promote beneficial microbial growth, suppress pathogenic bacteria, and maintain intestinal homeostasis. Recent studies have highlighted the potential of microecologics in influencing gut-brain interactions to alleviate depressive symptoms, positioning them as a promising adjunctive therapeutic approach for depression.

Probiotics, a key group within microecologics, have gained considerable recognition for their therapeutic potential in treating various conditions, including depression. They exert their effects primarily through the MGBA, influencing brain function by reducing systemic inflammation, repairing the intestinal barrier, and mitigating neuroinflammation (29). Several studies have identified specific probiotic strains with antidepressant potential (67). For example, *Lactobacillus* and *Bifidobacterium* species are among the most used strains in clinical settings due to their documented efficacy in modulating mood disorders (114, 242–244). However, results remain inconsistent across studies, with some reporting no significant improvement in depressive symptoms following probiotic administration (245, 246). This variability is likely due to factors such as strain specificity, multi-strain synergism, and the overall dosage (247, 248). An umbrella meta-analysis revealed that probiotic efficacy in treating depression is contingent upon both the dosage and duration of the intervention. Significant alleviation of depressive symptoms was observed only when the probiotic dose exceeded 10×10^9^ colony-forming units (CFUs) and the intervention lasted for more than 8 weeks (249). Additionally, certain microorganisms, particularly those difficult to culture, have demonstrated antidepressant effects. *Akkermansia* spp., for instance, has been shown to improve depressive behaviors in chronic stress models by restoring the balance of depression-related molecules such as corticosterone, dopamine, and BDNF (250). However, a gap remains between preclinical findings and clinical application, necessitating further research into the therapeutic potential of probiotics for depression.

Prebiotics, defined as selectively fermented compounds that confer health benefits, include substances such as fructooligosaccharides (FOS), galactooligosaccharides (GOS), inulin, and other soluble fibers (251). These compounds influence the gut microbiota indirectly by promoting the growth of beneficial bacteria. Evidence suggests that prebiotics may alleviate depressive symptoms by modulating the gut microbiota. For example, long-term administration of FOS and GOS has been shown to reduce depressive and anxious behaviors in animal models by enhancing acetate-producing bacteria (23). However, clinical evidence regarding the efficacy of prebiotics in depression is mixed. In a RCT involving 110 patients, a probiotic supplement significantly improved depressive symptoms compared to a placebo, while a prebiotic intervention had no substantial effect (252). This may be due to the indirect nature of prebiotics’ action, which works by modulating the gut microbiota rather than acting directly on the host. As a result, prebiotics are often used in conjunction with probiotics to optimize therapeutic outcomes. One study demonstrated that a complex probiotic formulation containing inulin was more effective than single supplements in improving psychological well-being and reducing inflammation in patients with depression (253).

The synergy between probiotics and prebiotics has led to the development of synbiotics, which combine both components to enhance therapeutic efficacy. Synbiotics offer potential benefits in treating depression by regulating gut microbiota composition and their metabolic products, reducing pro-inflammatory cytokines, and alleviating oxidative stress (254). A synbiotic formulation containing *Lactobacillus acidophilus*, *Lactobacillus casei*, *Bifidobacterium* species, and inulin has been shown to alleviate depressive symptoms in overweight or obese individuals (255). Similarly, a combination of probiotics (*L. acidophilus* T16, *Bifidobacterium* BIA-6, *B*. *lactis* BIA-27, *B*. *longum* BIA-4) and prebiotics improved depression severity and serum BDNF levels in patients undergoing hemodialysis (256). While synbiotics hold promise as a therapeutic intervention, their efficacy depends on identifying the optimal combinations of probiotics and prebiotics, highlighting the need for further research to refine formulations.

Postbiotics, comprising inactivated microbial cells and their bioactive metabolites, have emerged as another avenue for modulating gut-brain interactions. Postbiotics have been shown to alleviate depressive-like behaviors by influencing the gut-brain axis, offering a potential advantage over live probiotics in certain populations (257). For example, a postbiotic formulation derived from heat-inactivated Lactobacillus helveticus has been found to reduce anxiety and depressive-like symptoms in animal models while modulating dopamine and serotonin receptor expression in the brain (258). Given the potential risks associated with live bacterial administration, particularly in vulnerable populations such as critically ill patients, postbiotics may represent a safer and equally effective alternative for managing depression.

Collectively, microecologics, including probiotics, prebiotics, synbiotics, and postbiotics, represent a promising frontier in depression therapy. Although preclinical and early clinical studies show promise, further research is essential to fully understand their mechanisms of action and optimize their use for clinical applications in the treatment of depression.

### FMT for depression treatment

FMT represents an innovative therapeutic approach aimed at restoring the gut microbiota by transferring functional microbiota from a healthy donor’s feces into the recipient’s gastrointestinal tract. This concept, though relatively modern in clinical application, has ancient roots dating back to 4th-century China, where “Huang Tang”, a fecal suspension, was utilized to treat conditions such as severe diarrhea and food poisoning (259). By the Ming Dynasty, this practice expanded to include a broader range of gastrointestinal disorders, including constipation and abdominal pain (260). The therapeutic potential of fecal matter was also recognized in 18th-century Europe, particularly by Metchnikoff, who noted substantial health improvements from incorporating fermented foods into his diet (261). Over time, FMT evolved primarily as a treatment for various intestinal disorders, especially infectious conditions (262, 263), and its scope has since broadened to encompass non-infectious diseases, such as Parkinson’s disease, melanoma, non-alcoholic fatty liver disease, and type 2 diabetes (264–267).

Recent studies have increasingly focused on the role of FMT in managing psychiatric conditions, particularly depression. Depression, often associated with dysbiosis or an imbalance in the gut microbiota, has been linked to altered gut-brain interactions. The therapeutic potential of FMT in depression is under investigation, with studies revealing a notable ability of fecal microbiota to influence depressive phenotypes (268). Notably, research has demonstrated that fecal microbiota from depressed individuals or animal models can induce depression related phenotypes in healthy recipients. For instance, in an animal model, Flinders Resistant Line (FRL) rats exhibited marked depressive-like behaviors following FMT from patients diagnosed with MDD. This transfer of depressive symptoms was accompanied by significant shifts in the microbiota, notably an increase in Ruminococcaceae and *Lachnospira*, and a decrease in *Coprococcus*, suggesting a direct link between specific microbial taxa and depression-related phenotypes (269). In a similar vein, FMT from rheumatoid arthritis patients has been shown to induce depressive-like behaviors in mice, elevating pro-inflammatory cytokines, such as IL-6 and TNF-α, thereby highlighting the role of gut microbiota in modulating systemic inflammation and immune responses, which may contribute to the development of depression (270). In contrast, several studies have demonstrated the potential of FMT to alleviate depression-related phenotypes. For instance, in an animal model subjected to CUMS, FMT led to significant improvements in depressive behaviors, alongside an increase in Firmicutes abundance and a reduction in intestinal and neuroinflammation (271). Clinical trials further support the therapeutic potential of FMT in depression, particularly in patients with comorbid irritable bowel syndrome, a condition frequently associated with depression. These studies revealed that FMT administration not only reduced depressive symptoms but also promoted a shift in gut microbiota composition, including the reduction of pathogenic bacteria such as *Faecalibacterium*, *Eubacterium*, and *Escherichia coli*, which are often implicated in gastrointestinal and mood disorders (272).

Despite the promising therapeutic effects, the clinical application of FMT has been accompanied by reports of side effects, including transient diarrhea, constipation, abdominal pain, and low-grade fever (273). Moreover, the efficacy of FMT is influenced by various factors, including fecal dosage, infusion frequency, delivery route, and donor-recipient compatibility (274). Recent advancements in personalized precision FMT have introduced the potential of artificial intelligence (AI)-driven donor-recipient matching, which can significantly optimize the treatment process by tailoring the procedure to the unique microbiota profiles of both the donor and recipient. AI-based algorithms can analyze large-scale microbiome data to predict the most compatible donor-recipient pairs, thereby enhancing the therapeutic outcomes and minimizing adverse effects. These variables underscore the need for careful optimization of FMT protocols to maximize therapeutic benefits while reducing the risk of side effects. As the understanding of gut microbiota’s role in depression continues to expand, further research is essential to refine FMT techniques, integrate AI-driven precision matching, explore long-term effects, and determine the optimal use of FMT in the treatment of depression. The incorporation of AI-based typing and personalized microbiota-based transplantation approaches holds promise for making FMT a more reliable and effective treatment for patients suffering from depression.

## Conclusion & future perspectives

The gut microbiota has emerged as a central player in depression pathogenesis, with compelling evidence linking microbial dysbiosis to altered neuroimmune signaling, neurotransmitter metabolism, and HPA axis regulation through the MGBA. Recent advances in multi-omics technologies have identified specific microbial signatures (e.g., reduced *Faecalibacterium* and increased *Enterobacteriaceae*) and their neuroactive metabolites (e.g., diminished SCFAs, elevated LPS) as potential diagnostic biomarkers and therapeutic targets. While traditional antidepressants remain the cornerstone of treatment, emerging microbiome-based approaches, including probiotics, prebiotics, postbiotics, and FMT, have shown promise in alleviating depressive symptoms in both preclinical and clinical studies. Furthermore, innovative interventions such as next-generation probiotics engineered for targeted neuroactive compound delivery, phage-based microbial modulation, and AI-optimized FMT protocols, demonstrate transformative potential in the management of depression. Despite the promising advancements, several key challenges remain. First, the need to establish causal mechanisms using humanized gnotobiotic models and cutting-edge techniques like single-cell spatial metabolomics is crucial to unraveling the precise relationships between the microbiome and depression. Second, clinical translation is complicated by the heterogeneity of microbiome profiles, necessitating the development of standardized, multi-omics-defined microbial consortia for consistent therapeutic outcomes. Third, there is a pressing need to advance precision medicine approaches that integrate microbiome-host interactions with individual genetic, epigenetic, and lifestyle factors to ensure the most effective and personalized treatments. The integration of synthetic biology, including CRISPR-modified psychobiotics, nanotechnology (e.g., blood-brain barrier-penetrating microbial vesicles), and computational psychiatry is on the horizon, and could usher in a new era of microbiome-based depression therapies. Realizing the full potential of these approaches will demand interdisciplinary collaboration across fields like microbial genomics, neuroimmunology, and computational sciences. By bridging mechanistic insights with clinically actionable solutions, these innovations hold the potential to revolutionize the treatment of mental health disorders, offering a paradigm shift in the prevention and management of depression.
